# Identification of m^6^A-modified gene signatures in lung adenocarcinoma tumorigenesis and their potential role in drug resistance

**DOI:** 10.1007/s12672-025-02106-0

**Published:** 2025-03-25

**Authors:** Xiaomin Han, Qiang Ma, Ruyi Chang, Siyuan Xin, Guojun Zhang, Ruilong Wang, Yukun Wang

**Affiliations:** 1 Department of Pharmacy, Southern University of Science and Technology Hospital, Shenzhen, China; 2 Department of Pharmacology, School of Medicine, Southern University of Science and Technology, Shenzhen, 518055 Guangdong China; 3 Cancer Biology Institute, Baotou Medical College, Baotou, China; 4 No. 5 Railway Middle School, Baotou, China

**Keywords:** LUAD tumorigenesis, LASSO, WGCNA, Pearson correlation coefficient, m^6^A-seq, Drug resistance

## Abstract

**Background:**

Lung cancer is one of the most commonly diagnosed cancers. *N*^6^-methyladenosine (m^6^A) modification has a profound impact on RNA translation, splicing, transportation, and stability.

**Aims:**

This research aimed to identify and verify m^6^A-modified signatures for Lung adenocarcinoma (LUAD) tumorigenesis.

**Objective:**

Our previous mRNA-seq and m^6^A-seq data from 26 pairs of LUAD samples and tumor-adjacent normal tissues are used.

**Methods:**

Univariate Cox regression analysis and the least absolute shrinkage and selection operator (LASSO) analysis were used to estimate the significance of 37 collected m^6^A regulators. WGCNA was constructed to identify the genes correlated with LUAD tumorigenesis. Pearson correlation analysis between mRNA-seq and m^6^A-seq data was used to identify the m^6^A-correlation genes.

**Results:**

LASSO-Cox analysis identified 18 m^6^A significant regulators. The top 3 regulators, including METTL16, FTO, and SRSF10, and their downstream genes which were reported in the literature were analysed to confirm their role in LUAD tumorigenesis. Blue and brown coexpression modules were chosen as key modules for LUAD tumorigenesis. At last, we intersected Lasso-downstream genes, m^6^A-correlation genes, with blue or brown module genes. As a result, 56 m^6^A-modified gene signatures were obtained. Among them, AKAP9, PLXNB2, BRPF3, HPS4, EXOC7, and KLF6 have an inconsistent expression in protein and mRNA levels, probably due to m^6^A modification. In addition, these genes may be involved in regulating drug resistance.

**Conclusions:**

56 m^6^A-modified gene signatures for LUAD tumorigenesis were obtained from Pearson correlation analysis between mRNA-seq and m^6^A-seq data, along with LASSO and WGCNA analysis. Among them, AKAP9, PLXNB2, BRPF3, HPS4, EXOC7 and KLF6 play a crucial role in LUAD tumorigenesis in an m^6^A modification-dependent manner.

**Supplementary Information:**

The online version contains supplementary material available at 10.1007/s12672-025-02106-0.

## Introduction

Lung cancer is one of the most frequently diagnosed cancers and the leading cause of cancer-related deaths worldwide. In 2022, 1,060,600 new lung cancer cases and 733,300 cancer deaths are projected to occur in China [1]. Lung adenocarcinoma (LUAD) is the most common tissue histologic subtype of lung cancer, accounting for about 40% of lung cancer [2]. Up to 69% of patients with advanced non-small cell lung cancer (NSCLC) may have a potentially actionable molecular target, owing to the high prevalence of molecular aberrations in driver oncogenes observed in lung adenocarcinomas [3]. EGFR mutations occur in 10–20% of patients not of East Asian descent with NSCLC and in about 40% of Asian patients, mostly in adenocarcinoma, younger women, and girls. Environmental and personal habitats may have produced stronger sequence mutations such as EGFR, TP53, and KRAS, which then led to the development of the tumors [4].

Except for genetic mutation, Epigenetic variability such as DNA methylation, RNA modification, and histone modifications, have been widely reported to play an important role in tumorigenesis of lung cancer [5]. Different from the reversible epigenetic modification of DNA and histone protein to regulate gene expression, RNA methylation modification represents another layer of gene expression regulation [6]. *N*^6^-methyladenosine (m^6^A) modification, the most abundant epigenetic methylation of mRNAs and noncoding RNAs (ncRNAs), plays a pivotal role in regulating RNA translation, splicing, transport, and stability. This dynamic RNA modification is orchestrated by m^6^A regulators, including methyltransferases (“Writers”), RNA-binding proteins (“Readers”), and demethylases (“Erasers”) [7]. The methyltransferase-like (METTL) family is a diverse group of methyltransferases that can methylate nucleotides, proteins, and small molecules. The most well-characterized family members such as METTL3 and METTL14 dimerize to form an m^6^A RNA methyltransferase with established roles in cancer progression [8]. It has been reported that m^6^A RNA methylation regulators are strongly linked with the clinical and molecular characteristics of adenocarcinoma [9].

A considerable amount of studies that aimed to identify the prognostic signature based on m^6^A-related genes in lung adenocarcinoma have already been conducted [10, 11]. In previous studies, the analysis of Differentially Expressed Genes (DEGs) between LUAD samples and tumor-adjacent normal tissues was a conventional and widely adopted approach. However, these studies primarily relied on DEGs rather than individual expression matrices, resulting in the loss of substantial information and overlooking individual specificity and the complexity of tumorigenesis. In contrast, the current study utilized mRNA-seq and m^6^A-seq matrices instead of DEGs. We conducted Pearson correlation analyses between the mRNA-seq and m^6^A-seq matrices on an individual basis, identifying genes with high correlations that were speculated to be regulated by m^6^A modification. Subsequently, we hypothesized that certain m^6^A regulators and their downstream genes—identified through WGCNA and exhibiting correlations between mRNA-seq and m^6^A-seq—play critical roles in LUAD tumorigenesis.

## Materials and methods

### Patients and samples

**Fig. 1:**
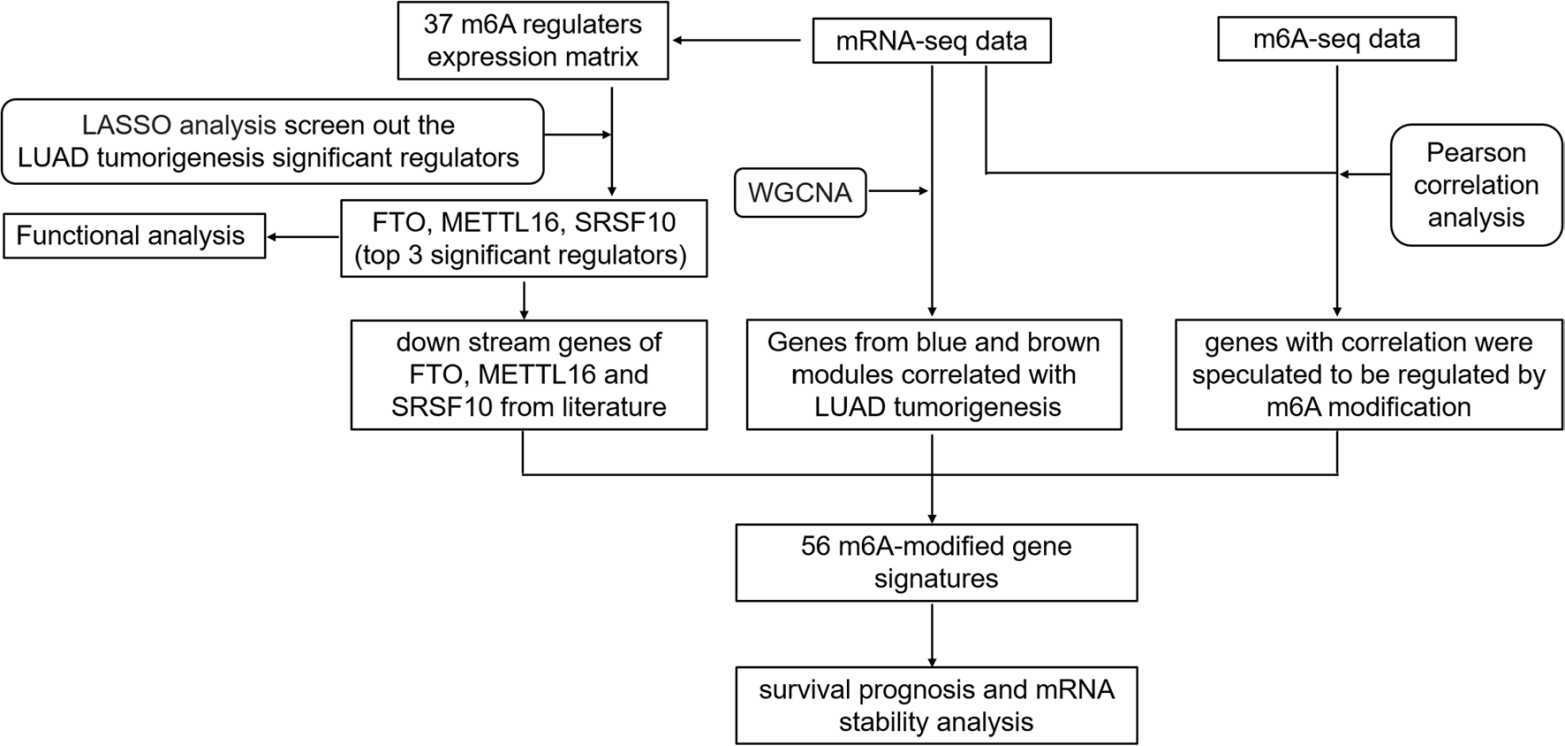
The flowchart delineates the steps for identifying and validating of 56 m^6^A-modified gene signatures

Figure 1 shows the research flowchart of this work. 26 pairs of LUAD samples and tumor-adjacent normal tissues were obtained from the South University of Science and Technology Hospital in our previous study. The detailed sample processing workflows, MeRIP Sequencing and RNA Sequencing were shown in our previous publication. This study was conducted according to the guidelines of the Declaration of Helsinki and approved by the ethics committee of the South University of Science and Technology Hospital. All participants provided written informed consent to participate in this study. The data are deposited in the GEO repository, with an accession number of GSE198288.

### Regression analysis of the m^6^A regulators

Univariate Cox regression analysis and the least absolute shrinkage and selection operator (LASSO) analysis were employed to evaluate the significance of 37 m^6^A regulators about patient survival. LASSO is a powerful feature selection technique that reduces overfitting by penalizing unimportant predictor variables. However, LASSO is highly sensitive to the choice of the regularization parameter (lambda), and an improper selection can lead to overfitting or underfitting. Additionally, LASSO assumes a linear relationship between predictor variables and the outcome, which may not always hold in complex biological systems. To address this limitation, we explored the impact of different regularization parameters on the results through sensitivity analysis and used alternative regularization techniques such as Elastic Net. Specifically, the “survival”, “dplyr”, and “glmnet” R packages were utilized to implement the methods. The analysis incorporated the tumor and non-tumor status of the samples, alongside gene expression profiles, to assess the survival outcomes associated with the expression levels of the m^6^A regulators. For the Univariate Cox regression analysis, we assessed the association between the expression of each of the 37 m^6^A regulators and patients’ overall survival (OS). The results were visualized in the form of forest plots, where the genes and their corresponding hazard ratios (HRs) with 95% confidence intervals (CIs) were displayed. This allowed for the identification of m^6^A regulators significantly associated with survival outcomes. To refine the list of candidate m^6^A regulators, we applied LASSO Cox regression, a technique designed to improve model prediction accuracy by performing variable selection and regularization. This method helps to identify a smaller set of the most predictive genes, effectively reducing overfitting. The penalty regularization parameter *λ* was determined through a tenfold cross-validation process, which minimizes the prediction error and ensures the stability of the model. The optimal *λ* value was selected based on the lowest mean cross-validation error. The genes selected through LASSO Cox regression were subsequently subjected to further validation to confirm their stability and reliability as prognostic biomarkers. Additionally, to better understand their clinical relevance in LUAD (lung adenocarcinoma) prognosis, the relationships between these selected genes and clinical features, such as tumor stage and grade, were investigated.

### Weighted correlation network analysis (WGCNA)

WGCNA is a powerful approach for constructing a scale-free topology. By using WGCNA, the correlations between the modules and clinical features can be systematically assessed. We conducted the WGCNA using the WGCNA shiny plugin in TBtools (version 2.041) [12]. A suitable *β* was calculated to satisfy the criterion of approximate scale-free topology. The correlation between co-expression modules and tumor or non-tumor status of samples was estimated. Module-trait relationships were calculated by Pearson correlation tests, and *p* < 0.05 was regarded as a significant correlation. WGCNA helps to identify gene modules with similar expression patterns and explore their association with clinical traits. However, it is sensitive to the choice of soft-thresholding parameters, and if the dataset is small or noisy, it may not capture all biologically relevant modules. To mitigate these limitations, we used other clustering techniques, such as hierarchical clustering, to confirm the robustness of the identified modules.

### Bioinformatics analysis

Signaling pathway analysis was performed at Metascape (https://metascape.org/) [13]. Gene ontology biological processes and KEGG pathway were included (*p* value <0.05). Protein–protein interaction (PPI) analysis was operated via STRING (https://cn.string-db.org/) (version 11.5) [14] and Cytoscape (https://cytoscape.org/) (version 3.9.1) [15]. Principal component analysis was conducted in TBtools (version 2.041). Kaplan–Meier Plotter (https://kmplot.com/analysis) and TNMplot (https://tnmplot.com/analysis/) were queried to elucidate the prognostic role and the expression of particular genes [16]. UALCAN (http://ualcan.path.uab.edu) is an interactive web portal to perform in-depth analyses of TCGA gene expression data, and at the same time, it enables the analysis of protein expression using public proteomic data [17].

## Results

### Identification of significant m^6^A regulators in LUAD tumorigenesis

In our previous study, mRNA-seq and MeRIP-seq analysis of tumor tissue and tumor-adjacent normal tissue from 26 LUAD patients was performed. In which it was demonstrated that m^6^A modification plays an important role in the progression and prognosis of LUAD [18]. To clarify the role of m^6^A regulators in our LUAD cohort, we collected 37 m^6^A regulator genes from previous studies as follows: 11 writers (METTL3, METL14, METL16, WTAP, RBM15, RBM15B, CBLL1, PCIF1 VIRMA, ZC3H13, and ZCCHC4), 23 readers (YTHDC1, YTHDC2, YTHDF1, YTHDF2, YTHDF3, HNRNPC, HNRNPA2B1, IGFBP1, IGFBP2, IGFBP3, CPSF6, EIF3A, ELAVL1, RBMX, FMR1, LRPPRC, NUDT21, NXF1, SETD2, SRSF3, SRSF10, TRMT112, and XRN1), and 3 erasers (FTO, ALKBH3, and ALKBH5) [19, 20].

**Fig. 2:**
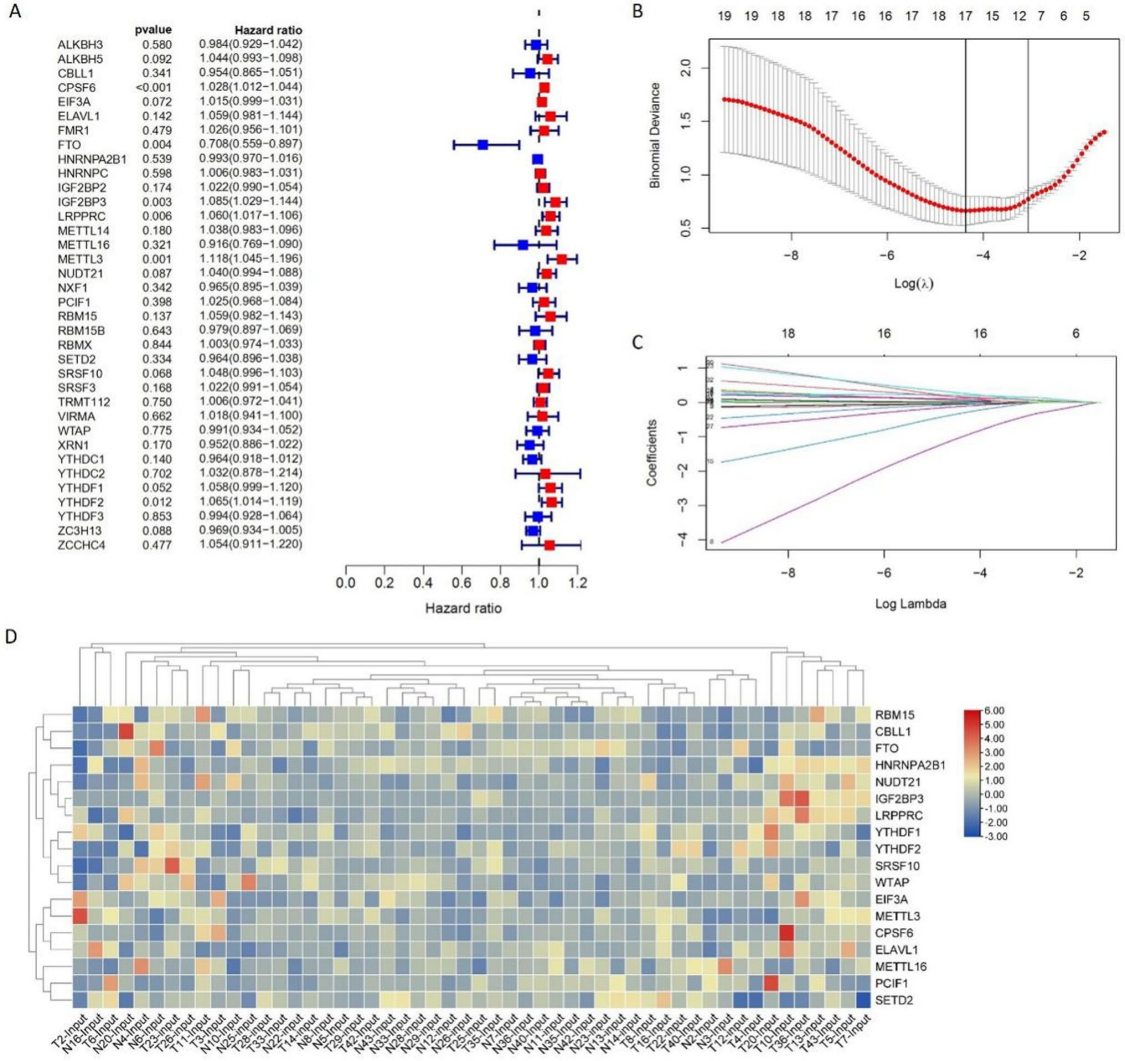
Construction of risk signature of 37 m^6^A regulator genes in the 26 LUAD samples. **A** Univariate Cox regression analysis of 37 m^6^A regulator genes for risk signature. **B** LASSO regression analysis reducing variants. **C** Coefficients of model-constructed genes obtained by LASSO. **D** Expression of 18 m^6^A regulator genes. Normalized as rows. Red indicating activation and blue indicating inhibition

Univariate Cox regression analysis and the least absolute shrinkage and selection operator (LASSO) analysis was used to screen out the significant regulators based on our data. Univariate Cox regression analysis showed that the expression of 37 m^6^A regulators is partly associated with the LUAD tumorigenesis (Fig. 2A). Then, LASSO-Cox analysis identified 18 significant regulators (Fig. 2B, C; Supplementary Table S1). The top 3 significant genes were FTO (−1.24), METTL16 (−0.43) and SRSF10 (0.34) (Fig. 2D). This result identified 18 significant m^6^A regulator genes in LUAD tumorigenesis.

### Functional analysis of the significant m^6^A regulators

**Fig. 3:**
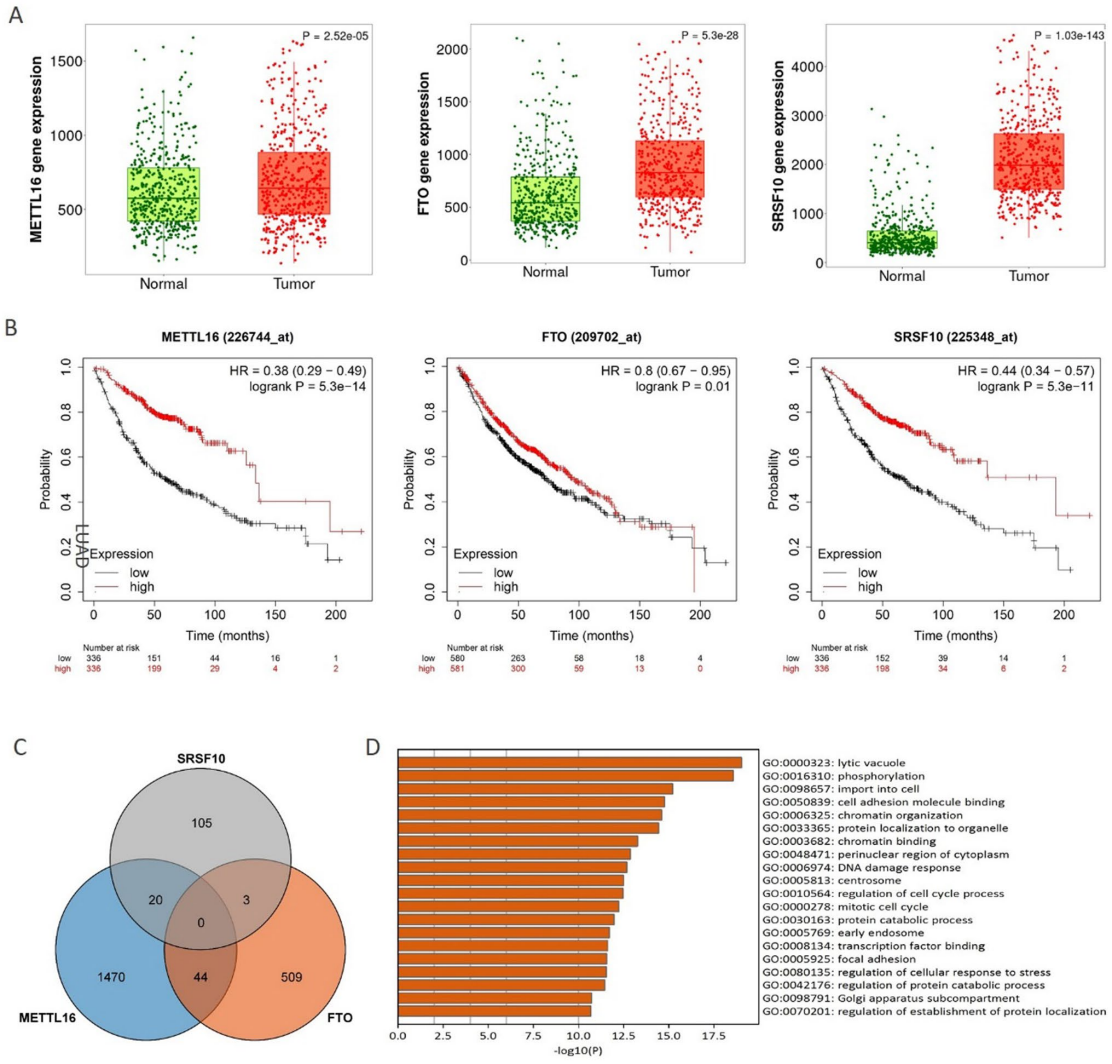
Tumorigenesis function of the top 3 Lasso genes. **A** Expression of METTL16, FTO, SRSF10 in LUAD tumor tissue and tumor-adjacent normal tissue via TNMplot. **B** Kaplan–Meier Plotter analysis of METTL16, FTO, SRSF10 in LUAD. **C** Venn diagram of downstream genes of METTL16, FTO, SRSF10. **D** Signaling pathway analysis of downstream genes of METTL16, FTO, SRSF10

To verify the function of 18 significant genes, we retrieved the expression of the top 3 significant genes in the TNMplot web portal. The results showed that the expression of METTL16, FTO, and SRSF10 was elevated in LUAD tissues (Fig. 3A). Kaplan–Meier Plotter analysis revealed that high expressions of these 3 genes were significantly correlated with increased overall survival in LUAD patients (Hazard Ratio = 0.38, 0.8, and 0.44, respectively) (Fig. 3B).

Considering that m^6^A regulators act by adding, removing, or recognizing m^6^A-modified sites of the downstream genes and alter important biological processes accordingly. We collected the downstream genes of METTL16, FTO, and SRSF10 as Lassso-downstream genes, including1534 hypomeythylation genes in METTL16 knockdown cells [21], 556 hypermethylation genes in FTO knockdown cells [22], and 148 differential genes in SRSF10 knockdown cells from the literature [23]. A Venn diagram of these downstream genes was constructed (Fig. 3C). Signaling pathway analysis revealed that these genes were enriched in lytic vacuole (GO:0000323), phosphorylation (GO:0016310), and import into cell signaling (GO:0098657) (Fig. 3D). These results validated the function of significant m^6^A regulator genes, in particular METTL16, FTO, and SRSF10.

### Identification of key modules in the LUAD tumorigenesis by WGCNA

**Fig. 4:**
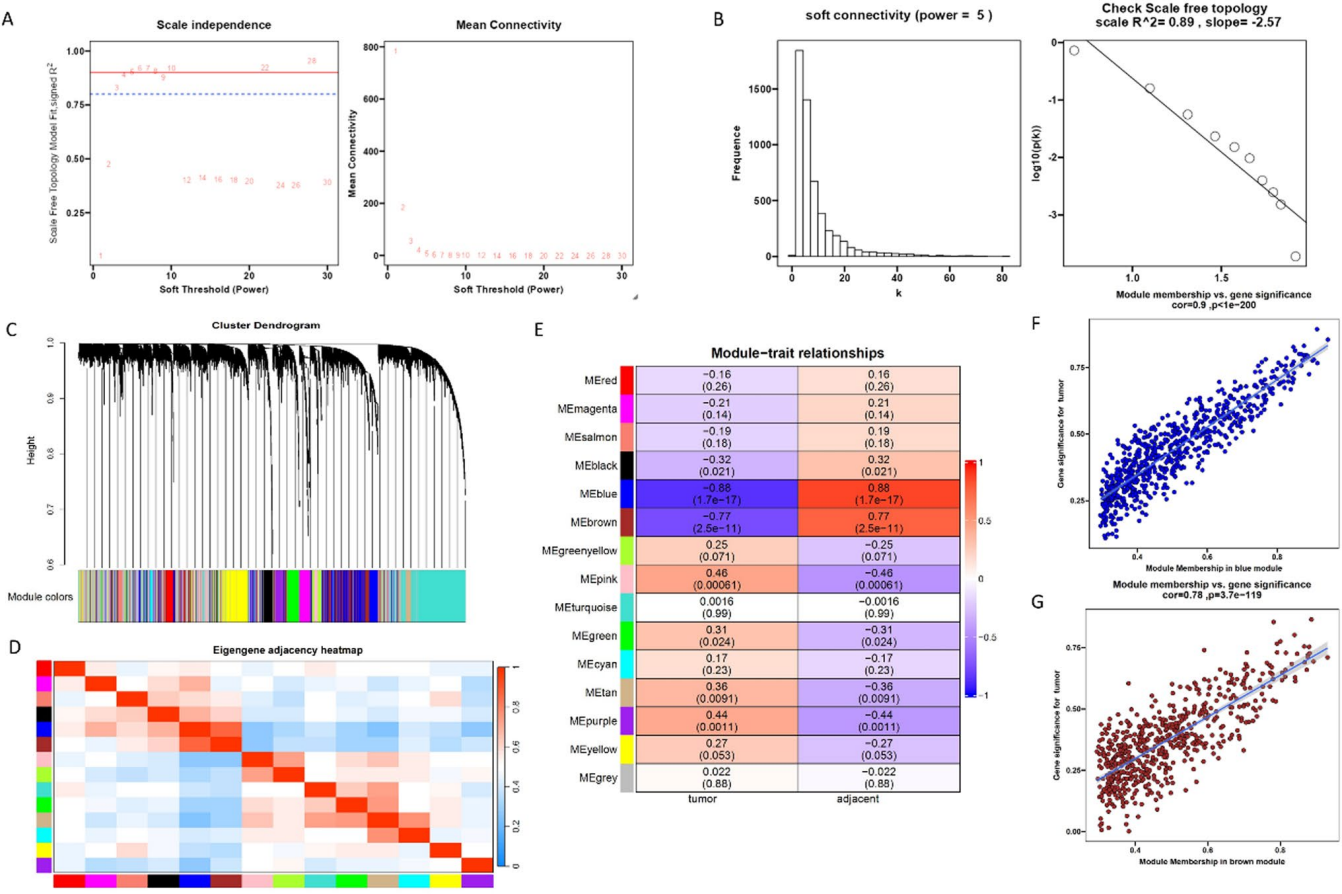
Screening of key modules for LUAD tumorigenesis by WGCNA. **A** Analysis of scale-free topology model fit index for different soft-thresholding powers (*β*) and the mean connectivity for soft threshold powers. **B** The frequence of soft connectivity and the check scale free topology by power *β* = 5. **C** Hierarchical clustering of the gene module based on the module eigengene. **D** Heatmap of module adjacency. The color bar represents the correlation between each module: the red indicates that the correlation between each module is high, and the blue indicates that the correlation between each module is low. **E** Heatmap of the correlation between consensus module eigengenes and tumorigenesis. The scatter plot of the top two important gene modules: blue module (**F**) and **G** brown module

We used the 26 pairs of tumor-adjacent normal tissues mRNA data to construct a weighted gene coexpression network, identifying the correlations between the genes and LUAD tumorigenesis. A scale-free network was generated by setting the soft threshold power to 5 (scale-free *R*^2^ = 0.89) (Fig. 4A, B). The clusters were obtained from the dendrogram by using the dynamic tree-cutting technique (Fig. 4C). A heat map was drawn to analyze and visualize the relationship of each module (Fig. 4D).

After the clinical sample traits were imported into the co-expression network, module-trait relationships were calculated. We found that the blue module (Pearson *r* = −0.88, *p* = 1.7e−17) and the brown module (Pearson *r* = −0.77, *p* = 2.5e−11) were negatively correlated with LUAD tumorigenesis. The pink module (Pearson *r* = 0.46, *p* = 0.00061) and purple module (Pearson *r* = 0.44, *p* = 0.0011) were positively correlated with tumorigenesis (Fig. 4E). The correlation values between module membership and gene significance were 0.9 and 0.78 in the blue and brown modules, respectively (Fig. 4F, G), which indicated strong relativity. Due to the correlation differences, the blue and brown modules were chosen as key modules for further study.

### Correlation analysis of mRNA-seq and m^6^A-seq

**Fig. 5:**
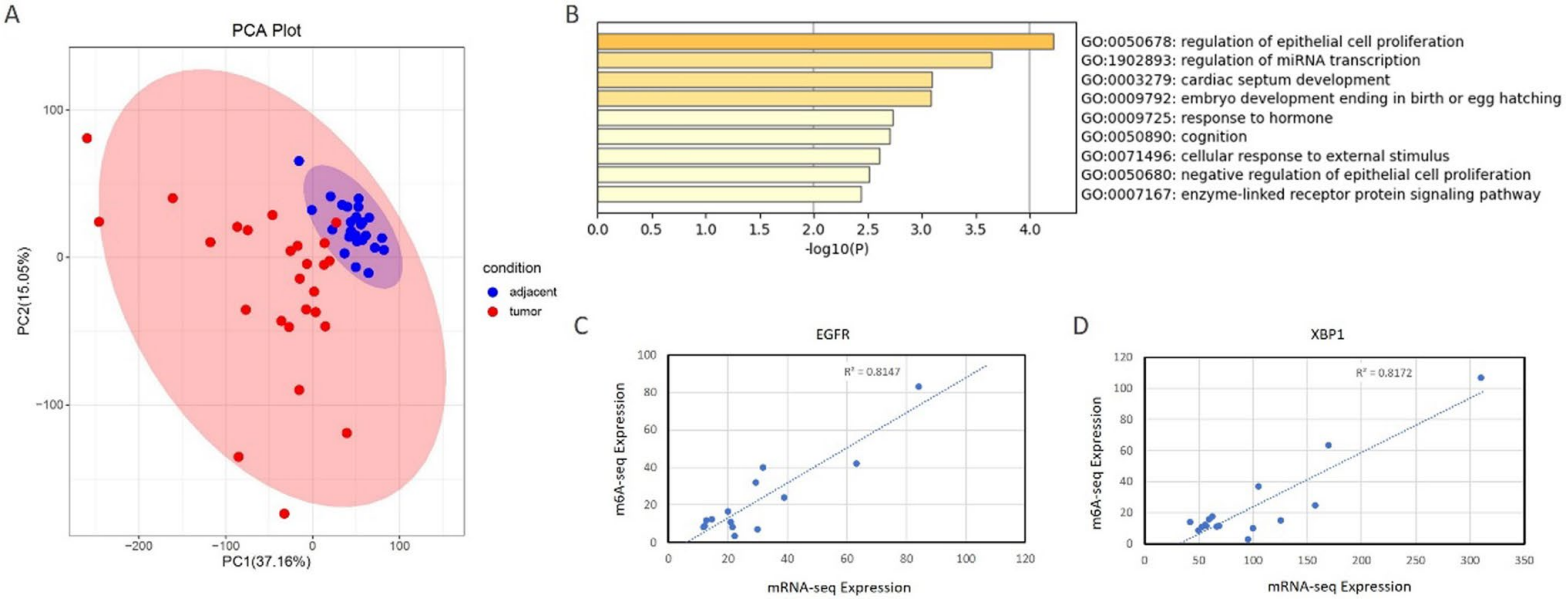
Correlation analysis of mRNA-seq and m^6^A-seq by Pearson correlation coefficient. **A** Principal components analysis plot for 55 top correlation genes of 26 pairs of samples. **B** Gene ontology analysis for 55 top correlation genes. **C**,**D** Scatter plots of Pearson correlations between mRNA-seq and m^6^A-seq of EGFR and XBP1

There is a strong linkage between m^6^A modification and mRNA stability. Thus, joint analysis of mRNA-seq and m^6^A-seq was carried out using the Pearson correlation coefficient among individual samples. We first identified 55 genes with a strong correlation whose absolute Pearson *r* value was 0.8–1 (Supplementary Table S2). The mRNA expression of these genes was used for principal component analysis (PCA). It was found that there were significant expression pattern differences between LUAD tissues and adjacent normal tissues (Fig. 5A). Signaling pathway analysis showed that these 55 genes were enriched in the regulation of epithelial cell proliferation (GO:0050678) and regulation of miRNA transcription (GO:1902893) (Fig. 5B). As an example, Pearson correlations between mRNA-seq and m^6^A-seq of EGFR and XBP1, belonging to the regulation of epithelial cell proliferation pathway, were shown using scatter plots (Fig. 5C, D). Meanwhile, studies have proved that EGFR and XBP1 are regulated by m^6^A modification [24, 25]. These results indicated that the Pearson correlation coefficient between mRNA-seq and m^6^A-seq can be used to identify the m^6^A-correlated genes.

### Identification of m^6^A-modified gene signatures for LUAD tumorigenesis

**Fig. 6:**
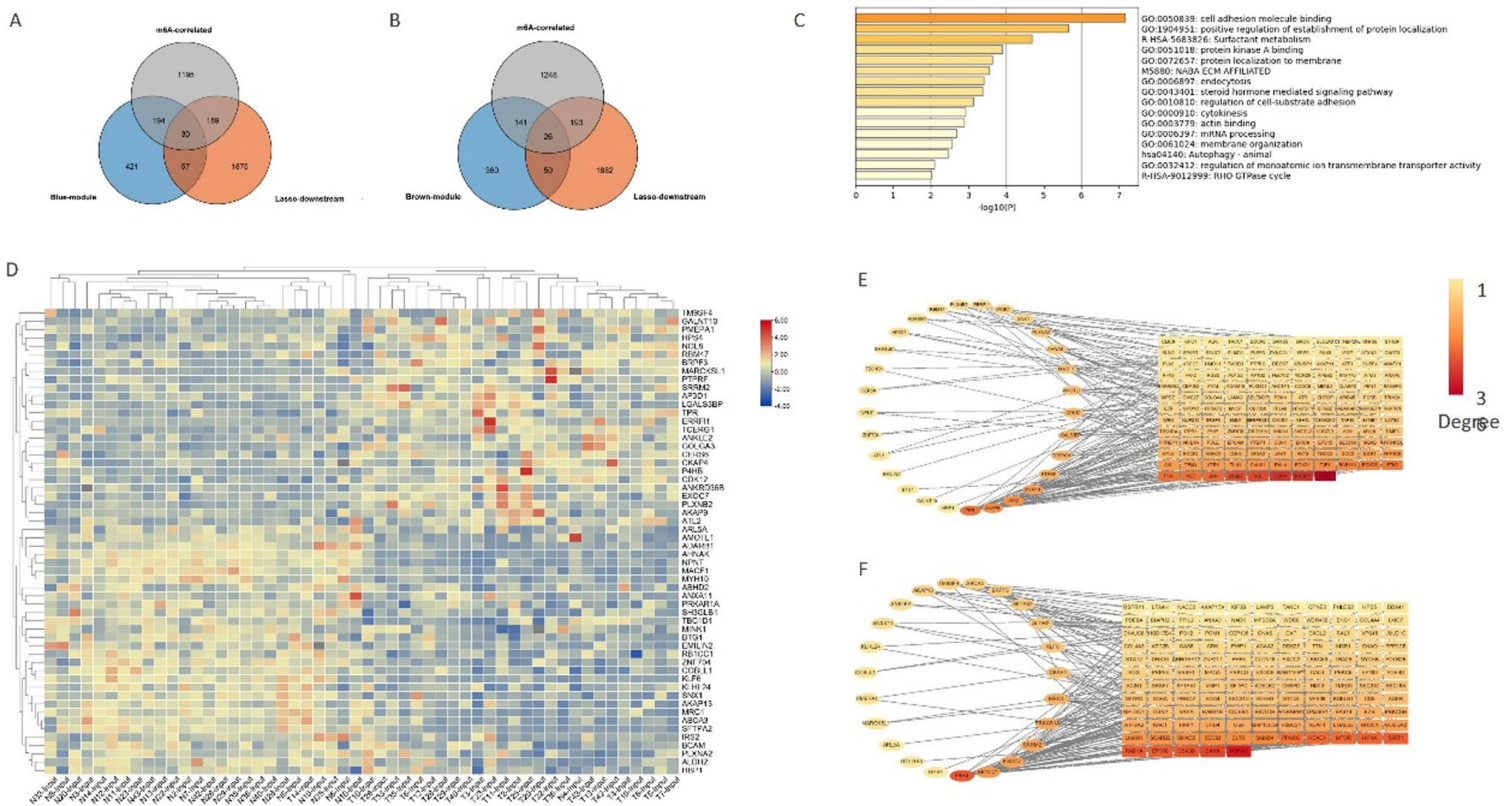
Identification of m^6^A-modified gene signature for LUAD tumorigenesis. **A**,**B** Venn diagram of Lasso-downstream genes, m^6^A-correlation genes, with blue or brown module genes from WGCNA. **C** Signaling pathway analysis of 56 m^6^A-modified gene signature for LUAD tumorigenesis. **D** Expression of 56 genes in 26 pairs of tumor-adjacent normal tissues. Protein–protein interaction of 56 genes in blue (**E**) and brown (**F**) WGCNA module. Ellipse indicate the 56 m^6^A-modified genes and squares indicate the other genes from their own module. The colour of the nodes represent the connectivity degree (red: high; yellow: low)

To identify the genes that were regulated by m^6^A modification and, at the same time, play a key role in LUAD tumorigenesis. We intersected Lasso-downstream genes, m^6^A-correlation genes, with blue or brown module genes from WGCNA. m^6^A-correlation genes contained 1608 genes with a correlation between mRNA-seq and m^6^A-seq (absolute Pearson *r* value was 0.3–1). Thus, we identified 56 m^6^A-modified gene signatures, and Venn diagram of these genes were constructed (Fig. 6A, B; Supplementary Table S3). The signaling pathway analysis showed that these genes were enriched in cell adhesion molecule binding (GO:0050839) and positive regulation of the establishment of protein location (GO:1904951) (Fig. 6C). The expression of these 56 genes differed between 26 pairs of tumor-adjacent normal tissues (Fig. 6D). Protein–protein interaction networks of 56 genes were constructed in blue and brown WGCNA modules, respectively (Fig. 6E, F). TPR and P4HB were the hub genes with the highest connectivity degree.

### Verification of the identified m^6^A-modified gene signatures in external data

**Fig. 7:**
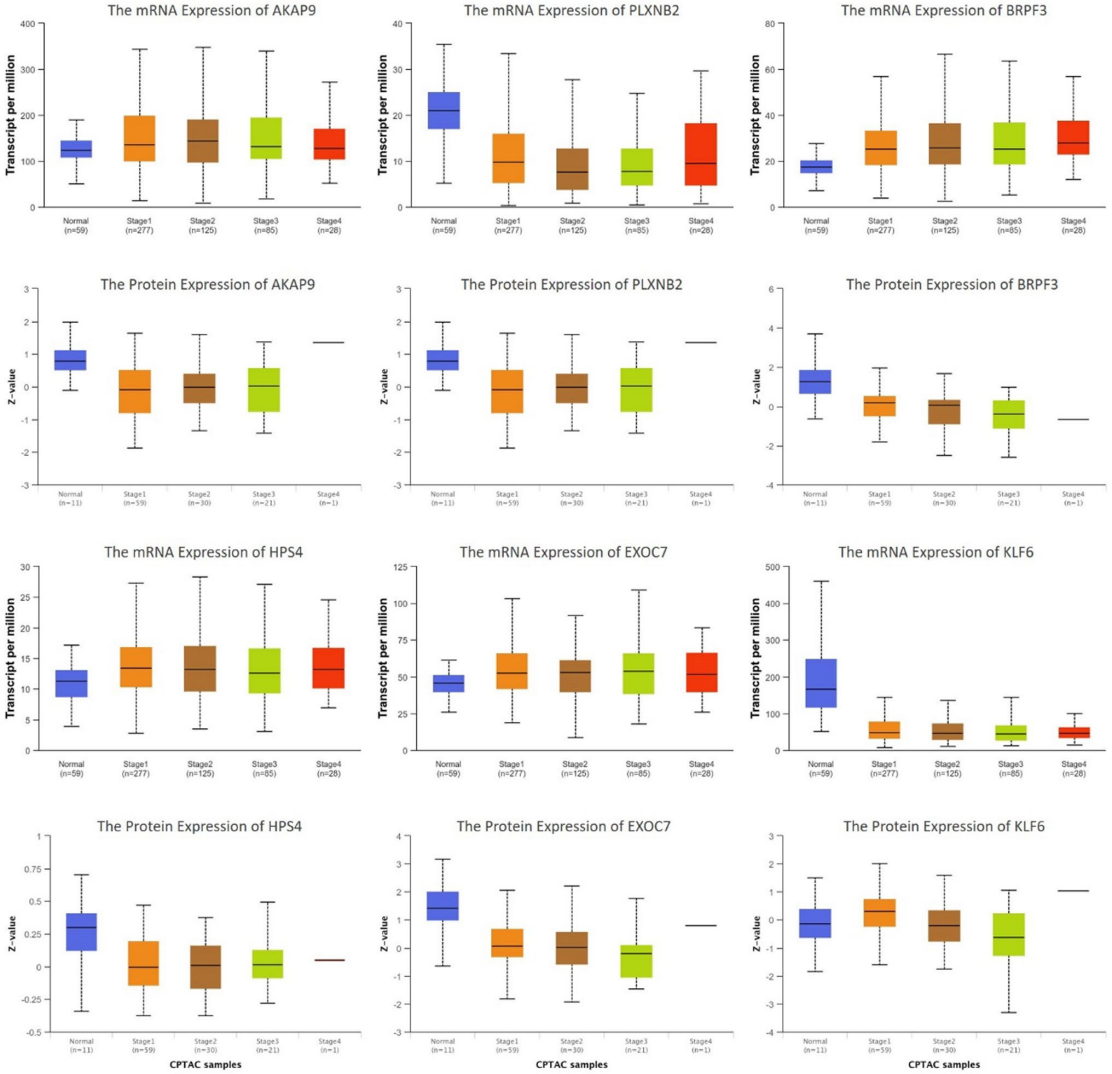
The protein and mRNA expression of m^6^A-modified gene signatures in UALCAN

**Fig. 8:**
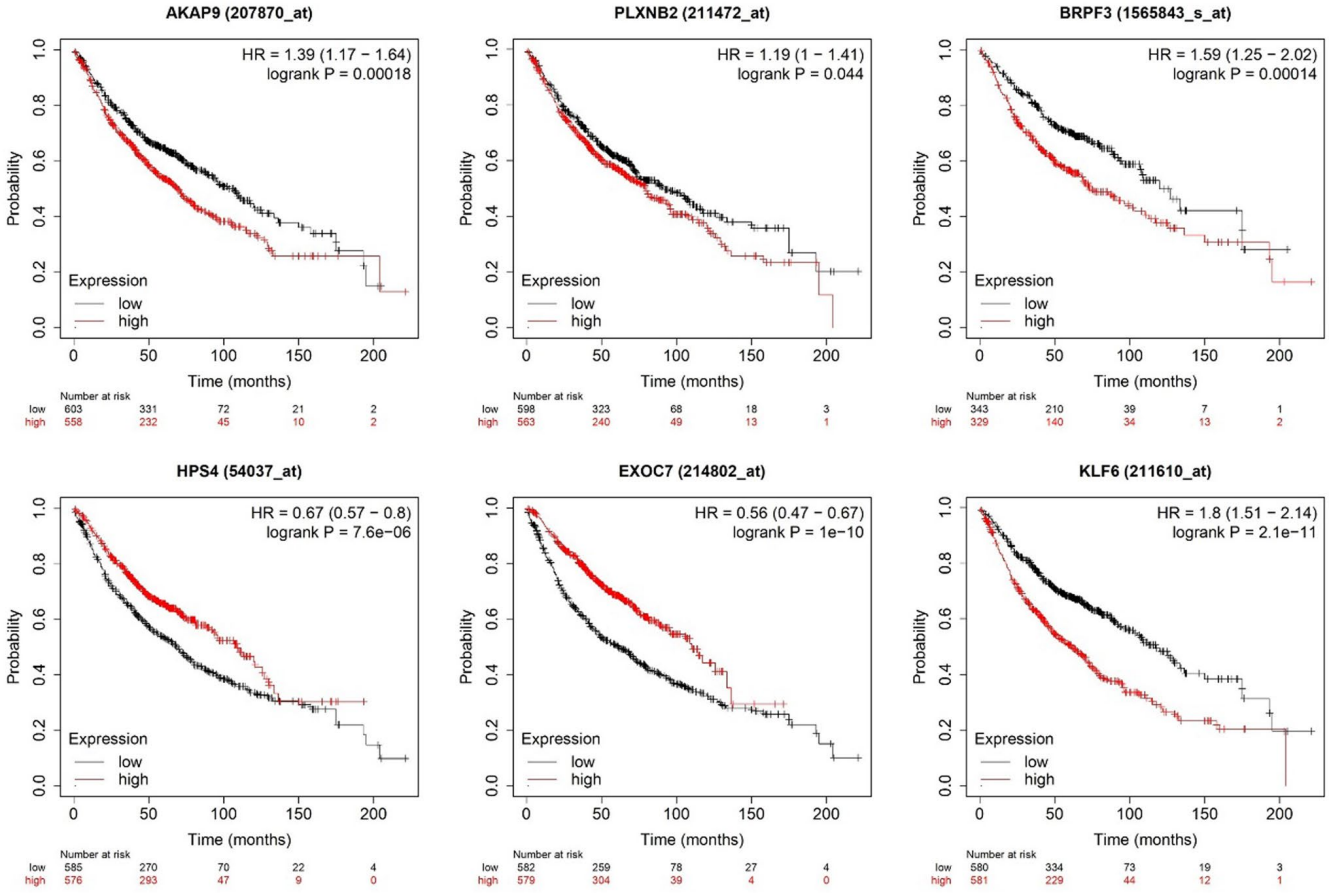
Kaplan–Meier Plotter analysis of m^6^A-modified gene signatures

To confirm whether there exists a difference between the protein expression and mRNA expression of these 56 genes due to the probability of m^6^A modification. We searched their protein and mRNA expression in UALCAN. The results revealed that the protein and mRNA expression of AKAP9, PLXNB2, BRPF3, HPS4, EXOC7 and KLF6 was not consistent (Fig. 7). The Kaplan–Meier Plotter analysis showed that mRNA expressions of these 6 genes were significantly correlated with increased overall survival in LUAD patients (Hazard Ratio = 1.39, 1.19, 1.59, 0.67, 0.56, and 1.8, respectively) (Fig. 8).

**Fig. 9:**
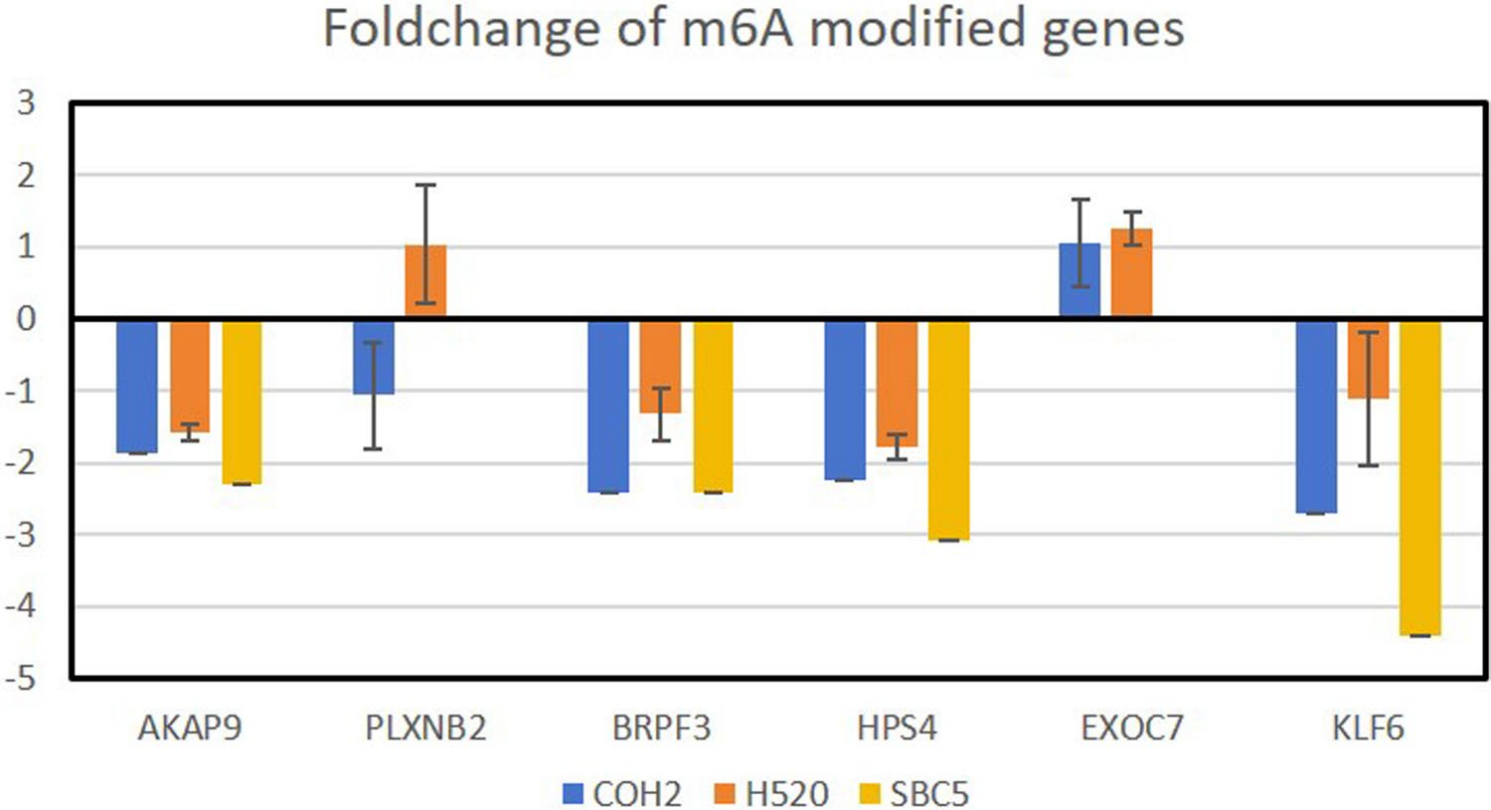
MRNA level of m^6^A-modified gene signatures after Actinomycin D treatment in COH2, H520 and SBC5 lung cancer cell lines

The stability of mRNA could be detected by using actinomycin D which could inhibit further transcription. In the previous study, we detected the mRNA level of these 56 genes in the presence of actinomycin D. Many of them had a diminished mRNA level, indicating poor stability in 3 lung cancer cell lines (Supplementary Table S4) [26]. Significantly, AKAP9, BRPF3, HPS4, and KLF6 have both poor mRNA stability and inconsistent protein expression (Figs. 7, 9). These results indicated that the 56 m^6^A-modified gene signatures function in LUAD tumorigenesis in a m^6^A modification-dependent way. In addition, AKAP9, BRPF3, HPS4, and KLF6 exhibit poor mRNA stability under actinomycin D treatment, and their protein expression is inconsistent with mRNA expression. This suggests that these genes may be involved in regulating drug resistance. Cancer cells may adapt to drug stress by altering the transcriptional and translational processes of these genes, thereby developing resistance to transcriptional inhibitors such as actinomycin D. Furthermore, these genes could be involved in the repair mechanisms, cell cycle regulation, or stress response of tumor cells, which play crucial roles in the adaptation and resistance to drug treatment.

## Discussion

*N*^6^-methyladenosine (m^6^A) is a crucial RNA modification that regulates various aspects of RNA metabolism, including stability, splicing, translation, and decay. m^6^A plays a significant role in tumorigenesis and progression in lung cancer. It regulates the expression of oncogenes and tumor suppressor genes, contributing to uncontrolled cell proliferation and resistance to apoptosis. m^6^A also influences the tumor microenvironment, affecting immune cell infiltration and metastasis, which can lead to immune evasion. Additionally, m^6^A modification is involved in drug resistance by modulating the expression of genes related to drug transport and metabolism. As a result, m^6^A has become a promising therapeutic target for enhancing treatment efficacy and overcoming resistance in lung cancer. WGCNA is a systems biology approach to characterize correlation patterns between genes. This analysis method is designed to find co-expressed gene modules and explore the associations between gene networks and phenotypes of interest. This approach is used to identify candidate biomarkers or therapeutic targets. We obtained two key modules that were highly correlated with LUAD tumorigenesis via WGCNA analysis (Pearson *r* = −0.88, −0.77, respectively) (Fig. 4). We speculate these genes play a key role in LUAD tumorigenesis. After that protein–protein interaction network of 56 m^6^A-modified gene signatures in blue and brown WGCNA modules was constructed to figure out the LUAD tumorigenesis (Fig. 6). TPR and P4HB were the hub genes with the highest connectivity degree. TPR (Translocated Promoter Region) is a prominent architectural component of the nuclear pore complex that forms the basket-like structure on the nucleoplasmic side of the pore [27]. The role of TPR is closely related to m^6^A modification.

Additionally, the pathway enrichment analysis revealed that these genes were involved in critical biological processes such as cell adhesion, protein localization, and epithelial cell proliferation. Targeting these pathways could lead to therapeutic interventions that specifically alter tumor cell behaviors, including migration and metastasis, which are key factors in LUAD progression. Thus, integrating m^6^A modification-related genes into clinical practice could aid in developing personalized treatment plans based on individual molecular profiles and improving the precision and effectiveness of LUAD therapies. Overall, our findings underscored the potential of m^6^A regulators and their downstream targets as therapeutic targets for LUAD, offering new opportunities for intervention in this highly aggressive form of lung cancer.

Identification of the Differentially Expressed Genes is conventional and universal which was at first used in previous articles including our earlier publication. However, these studies were based on DEGs rather than the Individual expression matrix, omitting a large amount of information and ignoring the individual specificity and the process of tumorigenesis. Our article was based on the mRNA-seq and m^6^A-seq matrix instead of Differentially Expressed Genes. We performed the Pearson correlation analysis between mRNA-seq and m^6^A-seq matrix individually, and genes with higher correlation which were speculated to be regulated by m^6^A modification were selected for the following research. We identified 55 genes with a strong correlation that the absolute Pearson *r* value was 0.8–1 (Supplementary Table S2). One of them, EGFR, has been proven to be a m^6^A modified target of METTL3 [24]. EGFR is a cell surface protein that binds to epidermal growth factor, thus inducing receptor dimerization and tyrosine autophosphorylation leading to cell proliferation [28]. EGFR is overexpressed at least fivefold across multiple epithelial cancers, including NSCLC, and thus has long been considered a therapeutic target [29]. The signaling pathway analysis showed that these 55 genes were enriched in regulating epithelial cell proliferation (GO:0050678) and miRNA transcription (Fig. 5B). These results validated that Pearson correlation analysis between mRNA-seq and m^6^A-seq could be used to identify the m^6^A-correlation genes.

To confirm 56 m^6^A-modified gene signatures for LUAD tumorigenesis. We queried the UALCAN web portal, and the results showed that the protein and mRNA expression of AKAP9, PLXNB2, BRPF3, HPS4, EXOC7, and KLF6 was not consistent (Fig. 7). The difference between the protein expression and mRNA expression is attributed to RNA translation, splicing, transportation, and stability. That is exactly what m^6^A modification impacts on. This m^6^A modification was regulated by significant m^6^A regulators such as METTL16, FTO, and SRSF10. It was reported that the expression of KLF6 was regulated by m^6^A modifications and has been recognized as a prognostic gene [30, 31]. The plasma protein level of PLXNB2 is associated with RNA modification-related SNPs [32]. These publications confirmed the reliability of our results. Importantly, the identification of m^6^A-modified gene signatures, including AKAP9, PLXNB2, BRPF3, HPS4, EXOC7, and KLF6, which are associated with m^6^A modification-dependent regulation in LUAD, thus laying a foundation for targeted treatment. These genes exhibit poor mRNA stability under actinomycin D treatment, indicating their potential role in drug resistance mechanisms. This suggests that these genes might be critical targets for overcoming chemoresistance in LUAD. By focusing on these m^6^A-modified gene signatures, novel therapeutic strategies can be developed to enhance the efficacy of existing treatments or overcome resistance to transcriptional inhibitors.

There are potential limitations in this study. Firstly, the study was based on a relatively small cohort of 26 LUAD patients for mRNA-seq and MeRIP-seq analysis. A larger and more diverse sample size could provide more robust and generalizable results. The small sample size might limit the statistical power and the ability to detect subtle associations between m^6^A regulators and LUAD tumorigenesis. Secondly, while the study identifies key m^6^A regulators (e.g., METTL16, FTO, and SRSF10) and downstream genes through bioinformatics analysis, the lack of experimental validation (e.g., knockdown or overexpression studies in cell lines or animal models) may raise concerns about the biological relevance of these findings. Thirdly, while the study associates m^6^A-modified gene signatures with LUAD tumorigenesis, it does not explore in detail the molecular mechanisms underlying the regulation of m^6^A modification on these genes.

## Conclusion

We collected 37 m^6^A regulators and performed LASSO Cox analysis to identify significant regulators. The top 3 regulators, including METTL16, FTO and SRSF10, and their downstream genes which were reported in the literature were analysed. WGCNA was constructed to identify the genes correlated with LUAD tumorigenesis. Pearson correlation analysis between mRNA-seq and m^6^A-seq was used to identify the m^6^A-correlation genes. At last, we intersected Lasso-downstream genes, m^6^A-correlation genes, blue or brown module genes from WGCNA, and 56 m^6^A-modified gene signatures were obtained. Among them, AKAP9, PLXNB2, BRPF3, HPS4, EXOC7, and KLF6 have inconsistent expression in protein and mRNA levels. Kaplan–Meier Plotter analysis revealed that mRNA expressions of these 6 genes were significantly correlated with increased overall survival in LUAD patients. AKAP9, BRPF3, HPS4 and KLF6 had poor mRNA stability after Antinomycin D treatment. These results verified their role in LUAD tumorigenesis in an m^6^A modification-dependent manner.

## Supplementary Information

Additional file1 (DOCX 30 KB)

## Data Availability

The datasets generated during and/or analyzed during the current study are available from the corresponding author upon reasonable request.
