# Supplementary material for: Identification of m6A-modified gene signatures in lung adenocarcinoma tumorigenesis and their potential role in drug resistance
Source: Discov Oncol. 2025 Mar 25;16:392. doi: 10.1007/s12672-025-02106-0 (PMC11937470; doi:10.1007/s12672-025-02106-0)
Supplement: Supplementary file 1 — Additional file1 (DOCX 30 KB) [file 12672_2025_2106_MOESM1_ESM.docx]

Supplementary Table S1. 18 significant regulators identified by LASSO-Cox analysis

| Gene Symbol | Coef |
| --- | --- |
| CBLL1 | -0.050798502 |
| CPSF6 | 0.074760332 |
| EIF3A | 0.003044277 |
| ELAVL1 | 0.084757728 |
| FTO | -1.236674877 |
| HNRNPA2B1 | -0.03524091 |
| IGF2BP3 | 0.114845905 |
| LRPPRC | 0.003175531 |
| METTL16 | -0.428524991 |
| METTL3 | 0.221553813 |
| NUDT21 | 0.095375878 |
| PCIF1 | 0.020746911 |
| RBM15 | 0.222790651 |
| SETD2 | -0.081210156 |
| SRSF10 | 0.338073651 |
| WTAP | -0.156717297 |
| YTHDF1 | 0.011113606 |
| YTHDF2 | 0.19932565 |

**Supplementary Table S2.** 55 genes with strong Pearson correlation

| Gene Symbol | Ensemble ID | Pearson correlation coefficient |
| --- | --- | --- |
| SHROOM4 | ENSG00000158352 | 0.862943862 |
| TRIM24 | ENSG00000122779 | 0.944979205 |
| FANCL | ENSG00000115392 | 0.901181037 |
| RASSF4 | ENSG00000107551 | 0.807714399 |
| PLXDC2 | ENSG00000120594 | -0.824767096 |
| RGMB | ENSG00000174136 | 0.909960622 |
| KDM5A | ENSG00000073614 | -0.801588487 |
| PSMC4 | ENSG00000013275 | 0.960254948 |
| HECTD1 | ENSG00000092148 | 0.803503885 |
| LTBP2 | ENSG00000119681 | 0.801620672 |
| WDR45B | ENSG00000141580 | 0.952839991 |
| LPGAT1 | ENSG00000123684 | 0.88521848 |
| RICTOR | ENSG00000164327 | 0.895664813 |
| RBBP8 | ENSG00000101773 | 0.813454625 |
| CLIC6 | ENSG00000159212 | 0.803757696 |
| SLIT3 | ENSG00000184347 | 0.964510503 |
| MACF1 | ENSG00000127603 | 0.848438169 |
| KHDC4 | ENSG00000132680 | 0.83609485 |
| AC068491.4 | ENSG00000279191 | 0.902208706 |
| EPM2AIP1 | ENSG00000178567 | 0.826558746 |
| ZNF529 | ENSG00000186020 | 0.836905713 |
| OTUD4 | ENSG00000164164 | -0.887224801 |
| NFIB | ENSG00000147862 | 0.869704826 |
| SULF1 | ENSG00000137573 | 0.943450398 |
| MMP28 | ENSG00000271447 | 0.971356561 |
| TAGLN | ENSG00000149591 | 0.822542752 |
| SLCO3A1 | ENSG00000176463 | 0.801082864 |
| AC037459.3 | ENSG00000253200 | 0.909025829 |
| MKL2 | ENSG00000186260 | 0.813639994 |
| ANKRD36C | ENSG00000174501 | 0.80282571 |
| DNAH11 | ENSG00000105877 | 0.959548303 |
| TNFRSF10B | ENSG00000120889 | 0.886190689 |
| TMEM57 | ENSG00000204178 | 0.836245257 |
| OLFML3 | ENSG00000116774 | 0.86062263 |
| LAX1 | ENSG00000122188 | 0.952261035 |
| LRRN4 | ENSG00000125872 | 0.861005953 |
| GOLGA1 | ENSG00000136935 | 0.872959696 |
| IYD | ENSG00000009765 | 0.870599678 |
| APOL6 | ENSG00000221963 | 0.837579501 |
| CYB5B | ENSG00000103018 | 0.817005479 |
| CHML | ENSG00000203668 | 0.873363324 |
| PLS3 | ENSG00000102024 | 0.808310822 |
| AC019117.1 | ENSG00000236039 | 0.801339985 |
| EGFR | ENSG00000146648 | 0.902588308 |
| AL662795.2 | ENSG00000280128 | 0.815833645 |
| DOCK2 | ENSG00000134516 | 0.879595646 |
| TMC5 | ENSG00000103534 | 0.851433267 |
| ABCA3 | ENSG00000167972 | 0.81435321 |
| XBP1 | ENSG00000100219 | 0.903995808 |
| IL13RA1 | ENSG00000131724 | 0.831330289 |
| TCERG1 | ENSG00000113649 | 0.845449982 |
| SCNN1A | ENSG00000111319 | 0.849606195 |
| DDX60 | ENSG00000137628 | 0.858584216 |
| KLHL11 | ENSG00000178502 | 0.826529653 |
| PTPRF | ENSG00000142949 | 0.835667846 |

**Supplementary Table S3.** 56 m6A-modified gene signatures

| Gene Symbol | Ensemble ID | Pearson correlation coefficient |
| --- | --- | --- |
| TCERG1 | ENSG00000113649 | 0.85, 0.48, 0.36 |
| ERRFI1 | ENSG00000116285 | -0.43, -0.38 |
| ADARB1 | ENSG00000197381 | 0.72 |
| AKAP9 | ENSG00000127914 | 0.58, 0.34 |
| MACF1 | ENSG00000127603 | 0.85, -0.56 |
| SNX1 | ENSG00000028528 | 0.43 |
| ZNF704 | ENSG00000164684 | 0.4 |
| HBP1 | ENSG00000105856 | 0.38, -0.39 |
| CDK12 | ENSG00000167258 | -0.36 |
| BTG1 | ENSG00000133639 | 0.34 |
| PTPRF | ENSG00000142949 | 0.34, 0.84 |
| MINK1 | ENSG00000141503 | 0.34 |
| AHNAK | ENSG00000124942 | -0.48, -0.45, -0.66, -0.45, -0.45 |
| ATL2 | ENSG00000119787 | -0.49 |
| AP3D1 | ENSG00000065000 | 0.43 |
| IRS2 | ENSG00000185950 | 0.73, 0.74 |
| GALNT10 | ENSG00000164574 | 0.66 |
| SH3GLB1 | ENSG00000097033 | 0.38 |
| NPNT | ENSG00000168743 | 0.49 |
| ANKRD36B | ENSG00000196912 | 0.72 |
| TPR | ENSG00000047410 | -0.48 |
| PLXNA2 | ENSG00000076356 | 0.34 |
| MYH10 | ENSG00000133026 | 0.6, 0.51 |
| PLXNB2 | ENSG00000196576 | 0.58 |
| AMOTL1 | ENSG00000166025 | 0.54 |
| LGALS3BP | ENSG00000108679 | 0.35 |
| EMILIN2 | ENSG00000132205 | 0.57 |
| CERS6 | ENSG00000172292 | 0.47 |
| TBC1D1 | ENSG00000065882 | 0.5 |
| RBM47 | ENSG00000163694 | 0.68, 0.44 |
| ANKLE2 | ENSG00000176915 | 0.52 |
| GOLGA3 | ENSG00000090615 | 0.39, 0.69 |
| SRRM2 | ENSG00000167978 | 0.33 |
| ANXA11 | ENSG00000122359 | 0.5, -0.35 |
| BCAM | ENSG00000187244 | 0.51 |
| PRKAR1A | ENSG00000108946 | 0.42 |
| ABHD2 | ENSG00000140526 | -0.31 |
| COBLL1 | ENSG00000082438 | 0.34 |
| BRPF3 | ENSG00000096070 | 0.33 |
| ABCA3 | ENSG00000167972 | 0.34, 0.38, 0.81 |
| HPS4 | ENSG00000100099 | 0.42 |
| KLHL24 | ENSG00000114796 | 0.38, -0.35 |
| AKAP13 | ENSG00000170776 | 0.38, -0.32 |
| ALDH2 | ENSG00000111275 | -0.4, -0.37 |
| RB1CC1 | ENSG00000023287 | -0.38 |
| MRC1 | ENSG00000260314 | 0.31 |
| PMEPA1 | ENSG00000124225 | 0.59, 0.58 |
| ARL5A | ENSG00000162980 | 0.39 |
| EXOC7 | ENSG00000182473 | 0.58 |
| CKAP4 | ENSG00000136026 | 0.51 |
| SFTPA2 | ENSG00000185303 | -0.58 |
| NOL8 | ENSG00000198000 | 0.66, 0.58 |
| TM9SF4 | ENSG00000101337 | 0.54, 0.7, 0.39 |
| MARCKSL1 | ENSG00000175130 | 0.39 |
| KLF6 | ENSG00000067082 | -0.4 |
| P4HB | ENSG00000185624 | 0.37 |

**Supplementary Table S4.** MRNA level of 56 m6A-modified gene signatures after Actinomycin D

| Cell line | COH2 | | H520 | | SBC5 | |
| --- | --- | --- | --- | --- | --- | --- |
| Gene Symbol | Fold change | P value | Fold change | P value | Fold change | P value |
| TCERG1 | -1.39 | 0.0926 | -1.73 | 0.081218 |  |  |
| ERRFI1 | -2.6 | 5E-07 | 1.12 | 0.50724 | -4.72 | 9.07E-09 |
| ADARB1 | -1.64 | 0.008 | -1.27 | 0.724161 |  |  |
| AKAP9 | -1.87 | 0.006 | -1.58 | 0.120328 | -2.3 | 1.58E-05 |
| MACF1 | -1.33 | 0.1117 | -1.73 | 0.193545 |  |  |
| SNX1 | 1.28 | 0.0709 | 1.27 | 0.221072 |  |  |
| ZNF704 | -2.08 | 2E-05 | -2.48 | 0.05684 |  |  |
| HBP1 | -3.81 | 2E-06 | -2.77 | 0.010808 | -6.63 | 7.05E-12 |
| CDK12 | -7.67 | 3E-15 | -2.85 | 0.00013 | -4 | 0.000186 |
| BTG1 | -4.92 | 4E-26 | -4.2 | 2.49E-06 | -8.94 | 6.87E-10 |
| PTPRF | 1.2 | 0.1373 | 1.47 | 0.084125 |  |  |
| MINK1 | -1.3 | 0.2902 | -1.13 | 0.730797 |  |  |
| AHNAK | -1.13 | 0.4864 | 1.07 | 0.820795 |  |  |
| ATL2 | -6.59 | 2E-17 | -3.2 | 3.07E-05 | -4.76 | 3.43E-10 |
| AP3D1 | -1.03 | 0.9237 | 1.01 | 0.695737 |  |  |
| IRS2 | -4.35 | 1E-16 | -1.54 | 0.26938 | -5.06 | 9.58E-08 |
| GALNT10 | 1.1 | 0.404 | 1.22 | 0.568298 |  |  |
| SH3GLB1 | -1.49 | 0.0295 | -1.32 | 0.398656 |  |  |
| NPNT | 1.16 | 0.1429 | -1.27 | 0.739299 |  |  |
| ANKRD36B | -1.32 | 0.1273 | 1.33 | 0.364547 |  |  |
| TPR | -1.99 | 0.0029 | -1.88 | 0.147203 |  |  |
| PLXNA2 | -2.17 | 5E-06 | -1.49 | 0.301315 |  |  |
| MYH10 | 1.05 | 0.6993 | -1.48 | 0.218926 |  |  |
| PLXNB2 | -1.06 | 0.7422 | 1.04 | 0.83479 |  |  |
| AMOTL1 | -4.17 | 1E-13 | -2.89 | 0.003785 | -3.2 | 1.41E-07 |
| LGALS3BP | 1.45 | 0.0155 | 1.32 | 0.161213 |  |  |
| EMILIN2 | -2.07 | 1E-04 | -1.49 | 0.221209 |  |  |
| CERS6 | -1.04 | 0.9287 | -1.17 | 0.68576 |  |  |
| TBC1D1 | 1.01 | 0.8361 | -1.04 | 0.966205 |  |  |
| RBM47 | -3.94 | 2E-09 | -1.4 | 0.104674 |  |  |
| ANKLE2 | -2.43 | 8E-06 | -1.64 | 0.111358 | -2.85 | 2.72E-07 |
| GOLGA3 | -1.22 | 0.1987 | -1.16 | 0.884894 |  |  |
| SRRM2 | -2.41 | 7E-07 | 1.36 | 0.776655 |  |  |
| ANXA11 | 1.17 | 0.2794 | 1.21 | 0.332404 |  |  |
| BCAM | 1.69 | 0.007 | 2.48 | 0.029835 |  |  |
| PRKAR1A | 1.13 | 0.2979 | -1.03 | 0.884975 |  |  |
| ABHD2 | -2.66 | 1E-04 | -1.14 | 0.604345 |  |  |
| COBLL1 | -2.08 | 0.0002 | -1.39 | 0.375033 |  |  |
| BRPF3 | -2.41 | 2E-06 | -1.32 | 0.36159 | -2.41 | 2.16E-05 |
| ABCA3 | 1.07 | 0.5685 | 1.56 | 0.087947 |  |  |
| HPS4 | -2.25 | 3E-05 | -1.77 | 0.165559 | -3.07 | 6.65E-07 |
| KLHL24 | -4.26 | 3E-14 | -2.33 | 0.004796 | -7.26 | 6.92E-06 |
| AKAP13 | -7.41 | 3E-23 | -4.26 | 1.16E-07 | -9.71 | 7.33E-19 |
| ALDH2 | 1.15 | 0.3688 | 1.34 | 0.170357 |  |  |
| RB1CC1 | -2.01 | 0.0004 | -1.91 | 0.048244 | -2.03 | 0.001366 |
| MRC1 | 1 | 0.2092 | 1.09 | 0.145246 |  |  |
| PMEPA1 | -3.46 | 0.0001 | -2.38 | 0.02244 | -2.58 | 0.001264 |
| ARL5A | -1.56 | 0.0184 | -1.69 | 0.117908 |  |  |
| EXOC7 | 1.06 | 0.5991 | 1.26 | 0.236577 |  |  |
| CKAP4 | 1.24 | 0.0979 | -1.46 | 0.174507 |  |  |
| SFTPA2 |  |  |  |  |  |  |
| NOL8 | -2.43 | 7E-05 | -1.11 | 0.882441 |  |  |
| TM9SF4 | -1.02 | 0.9251 | -1.17 | 0.703789 |  |  |
| MARCKSL1 | 2.69 | 2E-06 | 1.21 | 0.345965 |  |  |
| KLF6 | -2.71 | 2E-07 | -1.12 | 0.919201 | -4.41 | 5.37E-10 |
| P4HB | 1.48 | 0.0083 | 1.37 | 0.098783 |  |  |
